# 3800 Years of Quantitative Precipitation Reconstruction from the Northwest Yucatan Peninsula

**DOI:** 10.1371/journal.pone.0084333

**Published:** 2013-12-31

**Authors:** Alicia Carrillo-Bastos, Gerald A. Islebe, Nuria Torrescano-Valle

**Affiliations:** El Colegio de la Frontera Sur, Unidad Chetumal, Herbario, Chetumal, Quintana Roo, México; The Ohio State University, United States of America

## Abstract

Precipitation over the last 3800 years has been reconstructed using modern pollen calibration and precipitation data. A transfer function was then performed via the linear method of partial least squares. By calculating precipitation anomalies, it is estimated that precipitation deficits were greater than surpluses, reaching 21% and <9%, respectively. The period from 50 BC to 800 AD was the driest of the record. The drought related to the abandonment of the Maya Preclassic period featured a 21% reduction in precipitation, while the drought of the Maya collapse (800 to 860 AD) featured a reduction of 18%. The Medieval Climatic Anomaly was a period of positive phases (3.8–7.6%). The Little Ice Age was a period of climatic variability, with reductions in precipitation but without deficits.

## Introduction

The understanding of climate dynamics and the prediction of future changes require the identification of patterns of climatic change at different temporal scales [1]. To find these patterns, it is necessary to take into account continuous readings that go beyond instrumental measurements, which provide a temporally limited perspective.

Fossil pollen is a natural source of climatic information. Vegetation is subject to environmental conditions that favor certain species depending on their climatic preference. By calibrating modern pollen samples with climate variables, it is possible to reconstruct quantitative climatic parameters [2].

Precipitation is one of the most important and sensitive parameters of the tropical climate [3]. The reconstruction of precipitation is of great interest because it permits the validation of climatic change models by examining the patterns of the past, present, and future [4]. On the Yucatan Peninsula, the fossil pollen record has been analyzed in terms of vegetation change. However, the inferences concerning climate have been expressed only in qualitative terms (wetter/drier). In the Yucatan region, the variability in precipitation had an important influence on the cultural history of the Mayan civilization [5]. Previous studies on the peninsula have indicated that the droughts suffered during the Classic period contributed to the collapse of the Mayan civilization [6]–[9].

A quantitative estimate of the precipitation over the last 1500 years is available for the peninsula. The record is based on measurements of δ^18^O values in stalagmites from the Tzabnabh cave [8]. In that study, it was determined that precipitation decreased by 36–52% relative to modern precipitation during the multiple droughts at the end of the Classic period. Recently, based on quantitative analysis of high-resolution climate records, it was concluded that the droughts of the Terminal Classic period were due to the reduction in the frequency and intensity of summer storms [9].

The Yucatan Peninsula is influenced by a number of different components of the climate system, creating a zone of mixed climates. For this reason, it is necessary to investigate whether the changes that occurred in this region were similar in magnitude, frequency and time in order to understand climatic variability. These changes are of particular importance because studying the events of the recent past will permit us to foresee what may occur in the near future, as climatic forcing that operated during the late Holocene did not significantly change [10]. With the purpose of contributing to the knowledge of climatic variability of the Yucatan Peninsula, this study presents a reconstruction of the vegetation from the northwest corner of the peninsula and the first quantitative reconstruction of precipitation for the last 3800 years.

### Study Area

**Figure 1:**
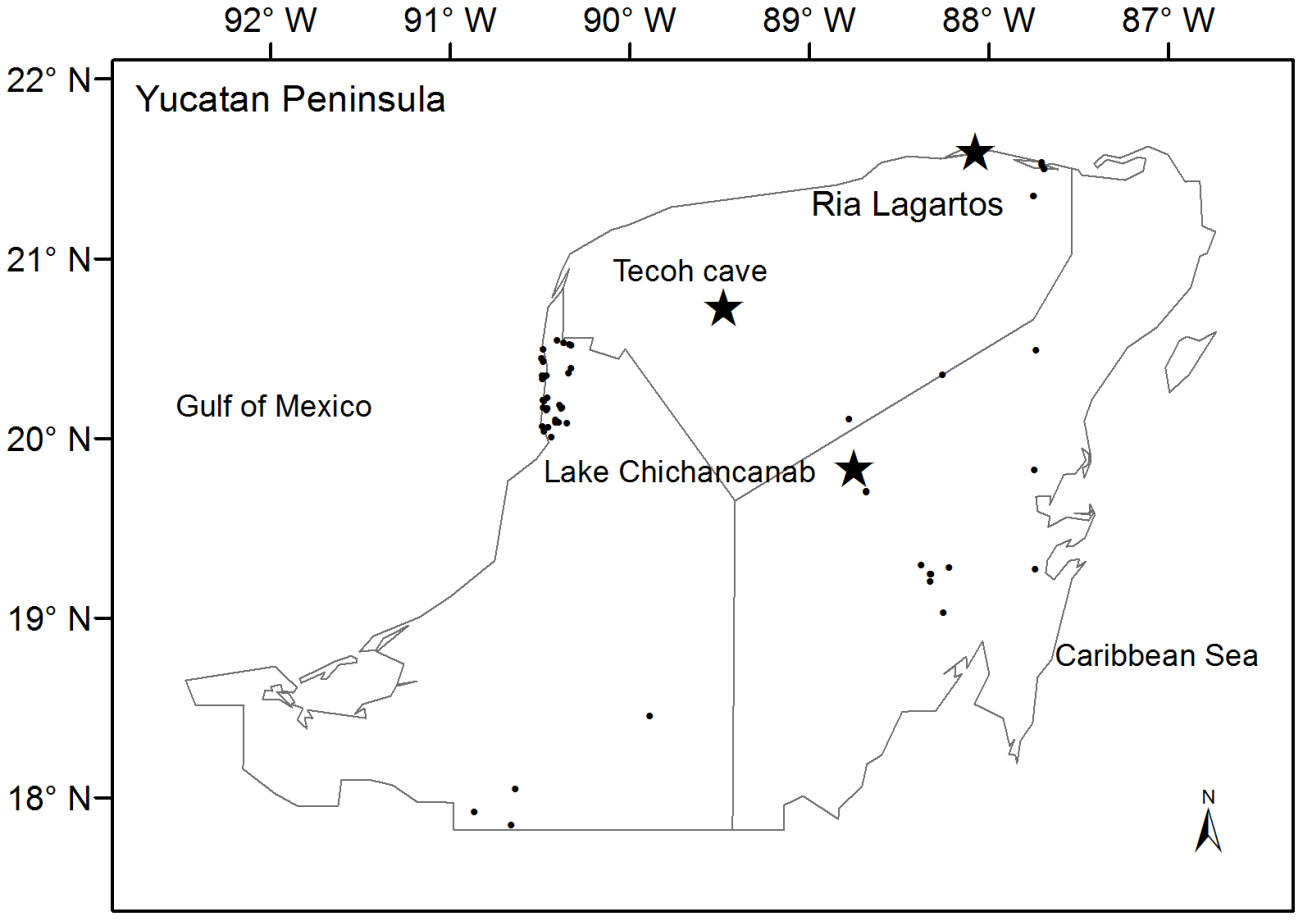
Map of area studied showing the sites mentioned in the discussion and the location of the modern pollen samples.

The study area is located in the northwest Yucatan Peninsula between the coordinates 21.5661N and 88.0865W in the biosphere reserve of Ria Lagartos (Figure 1). Because of its geographical position and proximity to the sea, the site is influenced by several different patterns of atmospheric circulation, such as the trade winds, polar air masses and the convection currents that provide precipitation [11]. The climate type is BSo(h’)w(x)iw, warm-semiarid and evaporation exceeds precipitation. The largest portion of the annual precipitation is received during the summer (62%), when the Intertropical Convergence Zone (ITCZ) and Bermuda Azores high-pressure cell move to the north and the trade winds intensify. The remaining precipitation (38%) occurs during the dry season from November to May. The influence of polar air masses occurs during the months of September to April [12]. The average temperature is 26°C [12], and the annual precipitation is 760 mm.

Several different vegetation regimes are found in the reserve: medium forest, dry forest, low forest, mangroves, coastal dunes and wetlands [11]. Vegetation near the coring site includes *Metopium brownei*, *Bursera simaruba*, *Haematoxylum campechianum*, *Conocarpus erectus*, *Plumeria sp*., and *Bravaisia sp*.

## Methods

### Calibration of Pollen Rain Data and Precipitation

#### Pollen data

**Figure 2:**
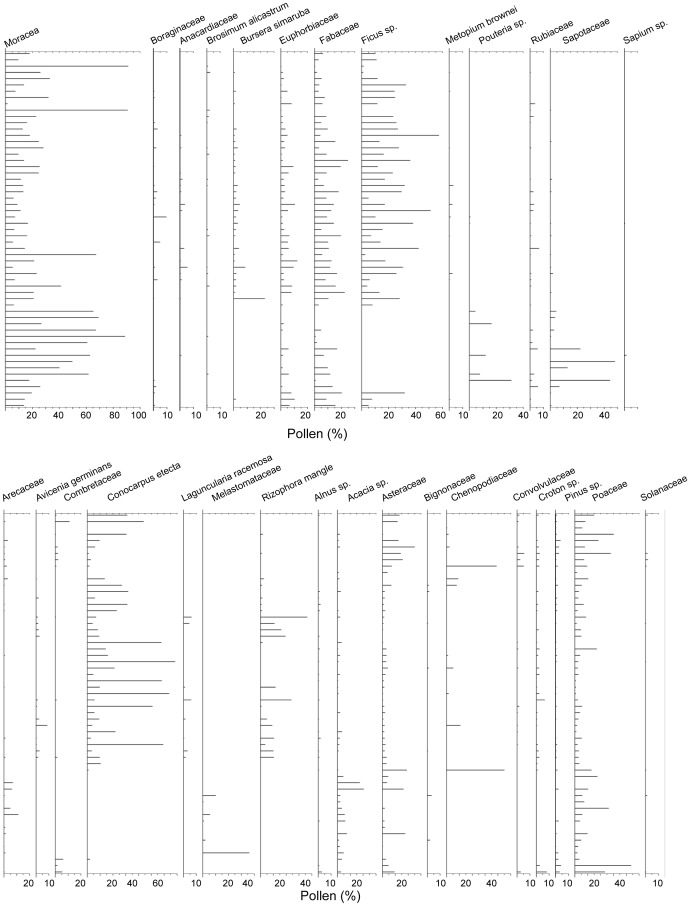
Percentage of taxa used in the calibration. The samples are sorted in ascending order with respect to the precipitation.

Samples of modern pollen rain were collected from different points of the Yucatan Peninsula (Figure 2), from a previous study [13] and present work. All are superficial sediment samples that were taken at a depth between 3 and 5 cm. At each site, 2–3 samples were taken and homogenized into one sample to avoid the effect of over-representation of local vegetation. A total of 57 samples were obtained.

The samples were treated with the standard techniques used for extraction and concentration: KOH, HCl, acetolysis [14], suspended glycerine gelatin (first study [13]) and an ultra-kit at 100% in this study. Tablets of *Lycopodium* were added to all the samples at the beginning of the treatments as an exotic marker. The pollen was identified with the help of the atlas developed by Palacios-Chavez et al. [15] and the palynological collection of Herbario-Ecosur (CIQR).

A minimum of 300 pollen of taxa belonging to pollen sum taxa were counted with an optical microscope with objectives of 400× and 1000×. Aquatic taxa and fungal spores were excluded from the pollen sum.

A total of 78 taxa were identified. To establish criteria of standardization and consistency in the data and to reduce bias, only taxa with percentages ≥1% and present in at least 5% of the samples were included [16]. Following this procedure, 30 species (Figure 2) were selected and the percentages were recalculated accordingly.

#### Precipitation data

Precipitation values were determined based on 65 meteorological stations on the Yucatan Peninsula from national weather services. At present, there are a total of 157 stations on the peninsula. However, only the 65 selected had at least 25 years of records during the period 1981 to 2010. The precipitation values for each sample of pollen rain were calculated by ordinary Kriging using the ArcGis 10 extension Geostatistical Analyst.

### Modern Pollen–precipitation Relationship

The first step in the quantitative reconstruction of precipitation is to determine if the relationship between the plant species and the environmental variable follows a linear or a unimodal model [2]. For this, it is necessary to estimate the gradient length of the environmental variable expressed in standard deviation (SD) units of biological turnover [2]. To estimate this, detrended canonical correspondence analysis (DCCA) [17] was conducted, using the software CANOCO 4.5 [18]. The following parameters were used: precipitation as the only predictor variable, detrending by segment, non-linear rescaling and pollen data transformed to the square root. During this analysis, four samples exhibited extreme values and as a result were discarded when constructing the transfer functions.

### Model of the Transfer Function

The length of the gradient of the environmental variable in SD units is an estimate of the behavior of the species along the length of this gradient [2]. If the gradient is small (2 SD units), the taxa behave monotonically through the gradient and the use of linear models of regression and calibration are appropriate [2]. In the DCCA, the length of the gradient obtained was <2 SD. Therefore, the transfer function was estimated using the method of partial least squares (PLS) regression [19], using software C2 1.7.2 [20].

PLS regression is a linear method that, by using inverse regression, calculates the transfer function that relates the modern pollen data to the environmental variable. This function can then be applied to fossil pollen data to reconstruct precipitation [2]. Evaluation of the prediction and selection of the best model with the smallest number of components are based on the combination of the values of maximum bias, root mean squared error (RMSEP), the coefficient of determination (r^2^) and the residuals, all four of which are based on the method of cross-validation by bootstrapping 1000 interactions. The model selected was a two component PLS regression.

The precipitation anomalies are calculated using the following formula: A = (R_i_ –Rm)*100/Rm, where A is the anomaly of every period, R_i_ is the estimated precipitation in the same period and R_m_ is the average of all recorded precipitation.

### Fossil Data

With a Russian corer, 50 cm sections of fossil sediment were obtained in a lagoon situated 5 to 7 km from the coastline. The total length of the core was 200 cm. The sediment was packed and transported to the palynology laboratory of Ecosur-Chetumal. The sediment was processed by physical and chemical treatments using the following protocol: treatment with HCl, filtration, digestion by KOH, acetolysis and mounted in Ultra-Kit at 100%. During the treatment with HCl, tablets of *Lycopodium* were added as an exotic marker. A total of 74 samples of fossilized pollen were obtained at an interval of 3 cm.

Taxa were separated by groups of ecological preference: tropical forest, disturbed taxa and mangrove. The pollen data are available in the Neotoma Database (http://www.neotomadb.org/uploads/data_subs/LAGARTOS.zip). The results obtained were graphed in a pollen diagram using the software TILIA 1.7.16 [21]. Pollen zones were determined by classification analysis based on the index of similarity using the tool CONISS, integrated into the same software package. The chronology was estimated by a linear regression applied to three radiocarbon dates (AMS) (Table 1) measured in the organic matter of the samples. The samples were analyzed in the laboratory of Beta Analytic Inc. in Miami, Florida. The radiocarbon dates were calibrated with the program CALIB 6.1.1 [22] using the IntCal09 calibration curve [23], rounded to the nearest decade and converted to calendar ages using 1950 AD as 0 cal yr BP.

**Table 1:** Radiocarbon ages (AMS) and sedimentation rates.

Sample code	Depth (cm)	Radiocarbon age(yr BP)	Calibrated age(cal yr BP)	Calendar age(BC/AD)	Relative area underprobability distribution (2σ)	Sedimentationrate (mm/yr)
Beta-317982	84.1	2160±30	2056–2185	106–235 BC	0.54807	0.40
Beta-325222	171.36	3020±30	3141–3338	1191–1388 BC	0.974839	0.78
Beta-325223	193.8	3130±30	3319–3409	1369–1459 BC	0.847747	1.80

Regression equation for chronology *Age = (depth+9.1809)/0.055* r^2^ = 0.95987.

### Ethics Statement

The samples are from locations where no specific permissions were required, and field studies did not involve endangered or protected species.

## Results

### Modern Pollen – precipitation Relationship

**Figure 3:**
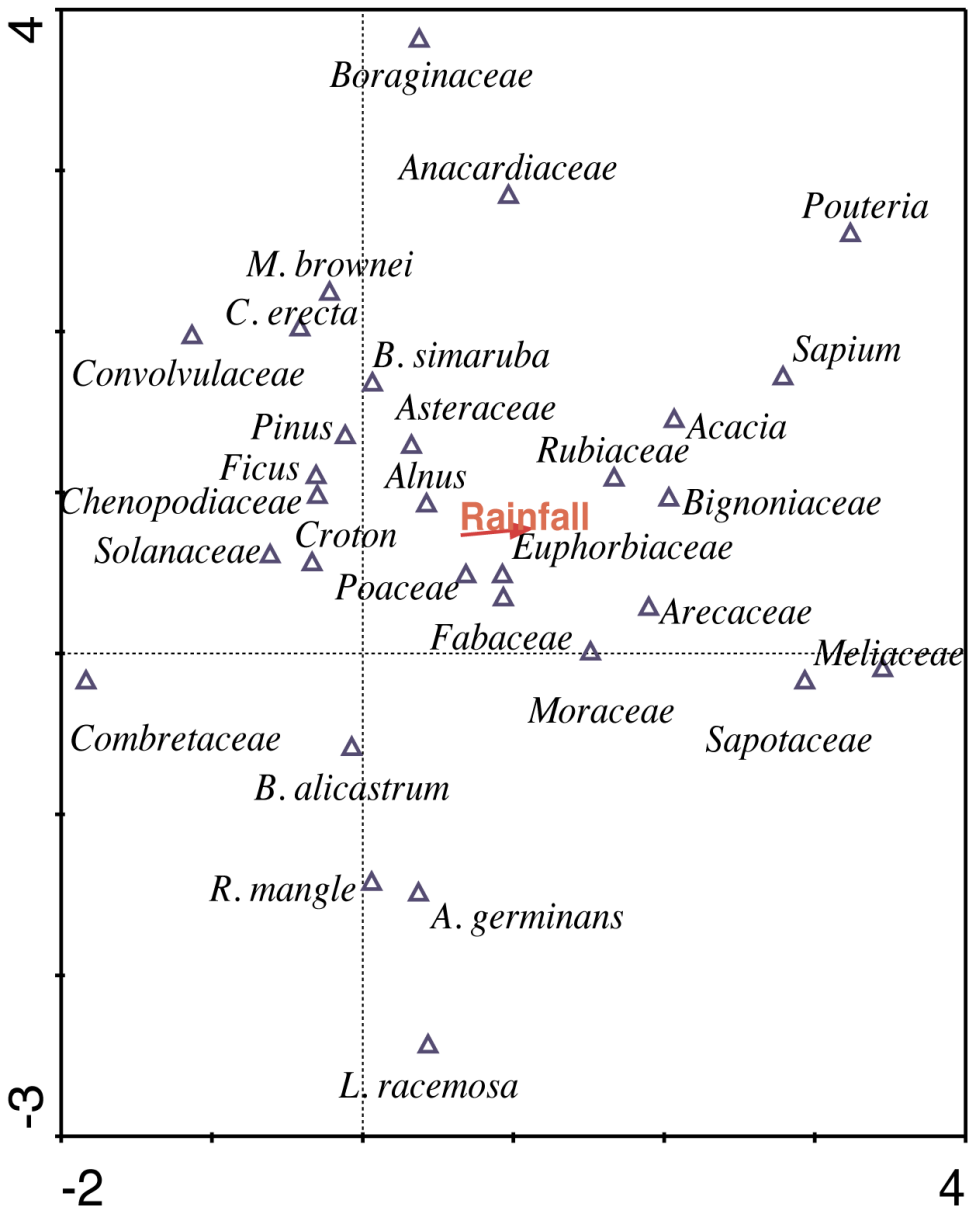
Ordination diagram results of DCCA.

In the DCCA, the length of the gradient was 1.69 SD. The correlation between the first ordination axis of the taxa and precipitation was 0.803, while the percentage of variance accumulated between taxa and the precipitation was 95.6%. The eigenvalue of the first ordination axis was 0.201. The ordination diagram showed separation of taxa in the first axis, which generated the correlation with the precipitation (Figure 3).

### Model of the Transfer Function

In the construction of the transfer function, a model with two components is used (Table 2). This model showed the lowest value of the RMSEP (131 mm) and a medium–high value for the coefficient of determination (0.703). The value of maximum bias (280.612) was not the smallest, but with this number of model components, the percentage of reduction in the RMSEP was 7.041% and the value of significance was 0.04.

**Table 2:** Model performance.

PLS component	RMSEP	r^2^	Maximum bias	% Change	t-test significance
1	140.97	0.486	348.176	…	…
**2**	**131.05**	**0.703**	**280.612**	**7.041**	**0.044**
3	133.21	0.735	255.696	−1.653	0.579
4	142.485	0.761	253.917	−6.956	0.984

Performance statistics of the four components of the PLS pollen-rainfall transfer function. Root mean squares error of prediction (RMSEP), coefficient of determination (r^2^), and maximum bias and reduction percentage in RMSEP given based on a bootstrapping cross-validation method. The select two-component model is shown in bold.

### Chronology and Reconstruction of the Vegetation

The resulting equation of the linear regression applied in conjunction with the calibrated ages (Table 1) produced, as a result, an age of 1850 BC at the bottom of the record.

The average calculated rate of sedimentation is 0.99 mm/yr. In the fossil pollen count, we identified 53 pollen taxa that belonged to 37 families.

**Figure 4:**
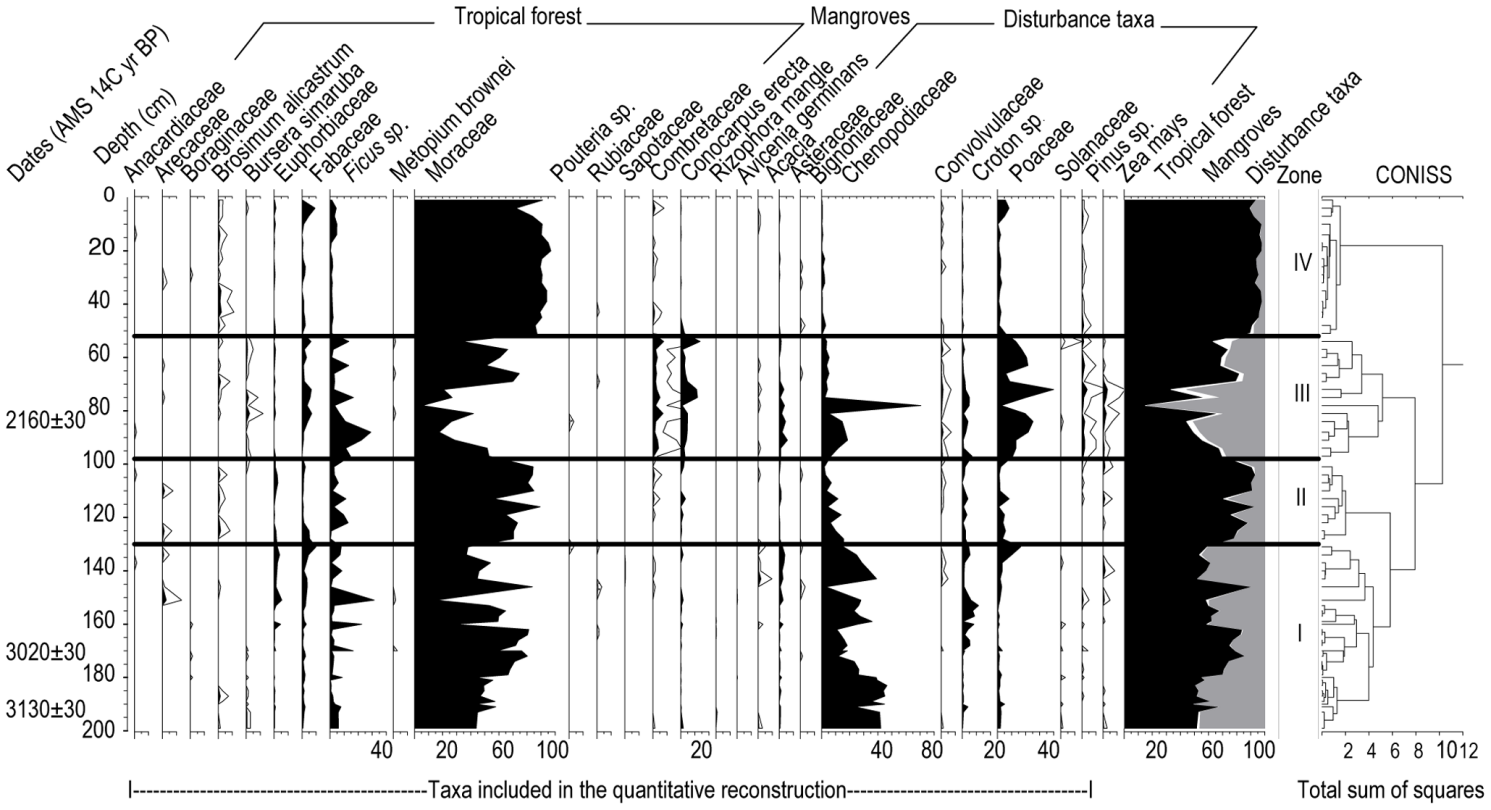
Diagram of fossil pollen.

Only those taxa composing ≥1% in at least 5% of the samples were used for the quantitative analysis (Figure 4). The curve of *Zea mays* is also represented because it gives valuable information about the presence of human activity. After applying the classification analysis to the percentages of pollen, four pollen zones were established, which represent the principal phases of vegetation change.

Zone I (200-130 cm, 1850-580 BC). In this zone, the main change is between taxa of the tropical forest and the disturbance taxa. The tropical forest oscillates between 50 and 90%, dominated by Moraceae (15–80%), followed by *Ficus sp*., Fabaceae and Euphorbiaceae. In the disturbed vegetation, the dominant taxa is Chenopodiaceae (40%) and, in lesser proportions, *Croton sp*., Asteraceae and Poaceae. Mangrove vegetation is scarce and does not reach 5%. The first record of *Z. mays* appeared around 1840 BC.

Zone II (130-97 cm, 580 BC-20 AD). The change during this zone is due to the decrease in disturbance elements (percentages ≤20%) and the increase in forest taxa (70–89%). *Pouteria sp*. and Rubiaceae are present. *Brosimum alicastrum* is present almost continuously, and Moraceae increases (values ≥58%). There is also a slight increase of mangrove (values ≤3.3%), especially *Conocarpus erectus*. *Z. mays* appears discontinuously.

Zone III (97-57 cm, 20–750 AD). This zone is marked by an increase of disturbance vegetation and mangroves and decrease of forest taxa. *B. alicastrum* is not present at the start of this zone, and *Bursera simaruba* appears, while Moraceae drops to 10%. *Z. mays*, Asteraceae and *Pinus sp*. are continuous throughout and in greater percentage than in the previously mentioned zones. Chenopodiaceae and Poaceae predominate in this vegetation zone. The presence of *C. erectus* is continuous and in percentages between 2 and 12%.

Zone IV (57-0 cm, 750 AD - present). Percentages of disturbed taxa are low, such as Chenopodiaceae (≤2%), Poaceae (≤8%), Asteraceae (≤1%), and *Croton sp*. (≤1%). *Z.mays* does not appear, and *Pinus sp.* are found. Moraceae is the dominant element (≥70%). *Ficus sp*. are lower. *B. simaruba* is not present, while *B. alicastrum* is present continuously. The curve of *C. erectus* is discontinuous.

### Quantitative Precipitation Reconstruction

**Figure 5:**
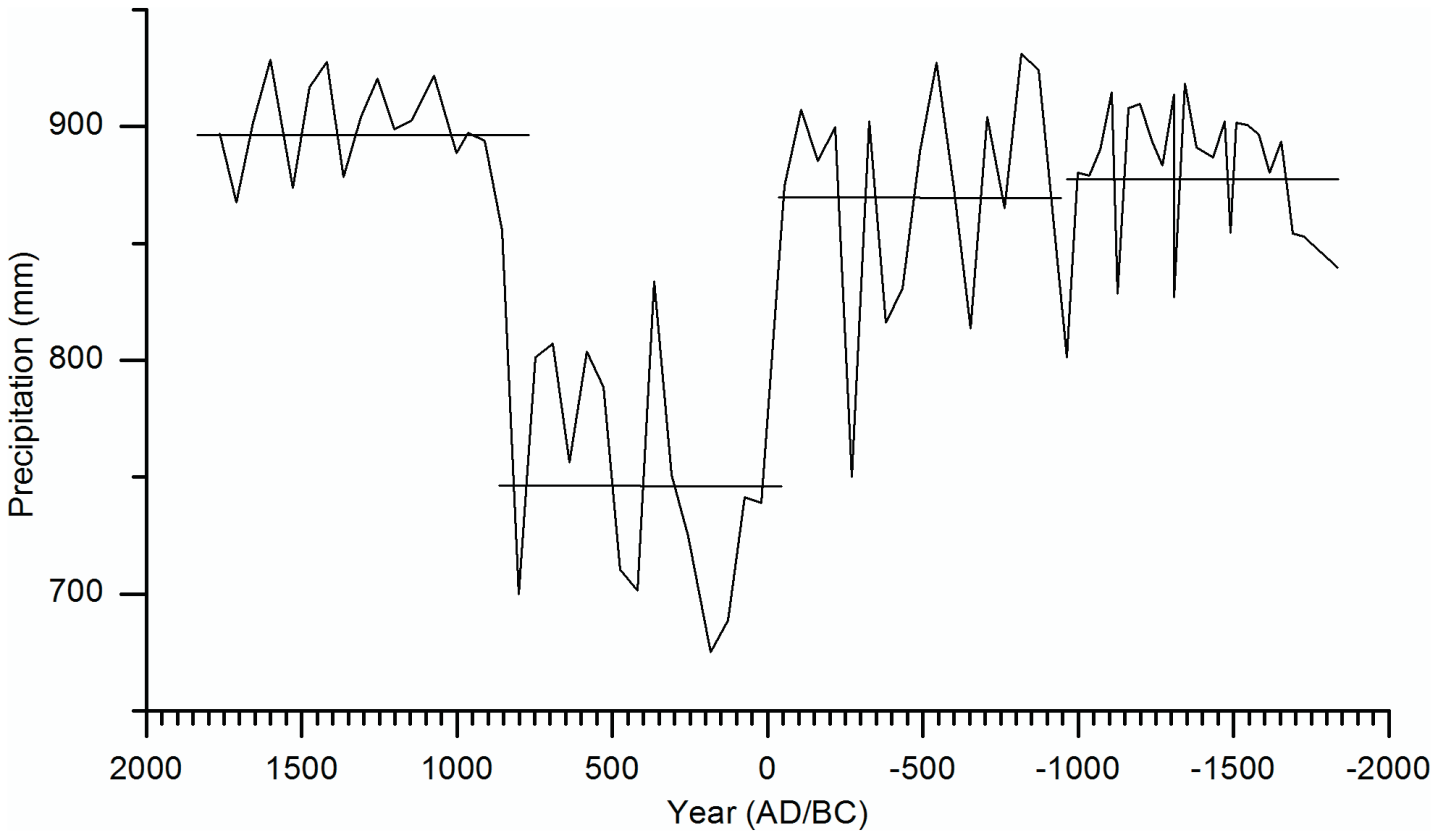
Inferred precipitation from fossil pollen. The horizontal lines indicate the averages for every phase.

The application of the transfer function to the pollen data of Ria Lagartos permits the reconstruction of precipitation variability during the last 3800 years. The average precipitation for this period is 850 mm with a SD of 66 mm. Four phases of change are detected: the first from 1850-1000 BC with an average of 883 mm, the second from 1000-50 BC with an average of 870 mm, the third from 50 BC-500 AD with an average of 748 mm and the fourth from 500–1770 AD with an average of 898 mm (Figure 5). Upon calculating the anomalies of precipitation, it appears that the greatest deficit occurred during the third phase. The years 180, 420 and 800 AD feature negative anomalies of 21% in the first extreme drought and 18% in the other two droughts.

However, the surplus did not even reach 9%. There were two surpluses in the second phase of change (820 and 540 BC) and two in the fourth phase (1420 and 1600 AD).

## Discussion

### Modern Pollen – precipitation Relationship

The longitude of the gradient obtained with the DCCA was low (<2 SD). Therefore, the relationship between taxa and precipitation has a monotonic relationship. From ecological studies, it is observed that species abundance exhibits a unimodal behavior with respect to an environmental variable [2]. Each taxon grows best at an optimum value of the environmental variable and is unable to fully develop at higher or lower values [24]. The range captured by the values of precipitation is most likely only a fraction of the unimodal curve. This explains why the taxa in this study showed monotonic and unimodal behavior. For the same reason, it is probable that the ordination diagram will show a short vector for precipitation. However, the diagram can show that the first axis of ordination separates the majority of the groups and their correlation with precipitation was 0.803.

Along this axis, the referred taxa act as indicators of humidity. Conserved vegetation in the paleoecological studies of the region [25]–[28] are ordered in the direction of the precipitation vector (e.g., Moraceae, Meliaceae, Sapotaceae, *Pouteria sp*., Rubiaceae and Sapindaceae). Taxa associated with less humid conditions and with disturbance vegetation are grouped on the opposite side of the graph (e.g., Chenopodiaceae, *Croton sp*., Asteraceae, Solanaceae, Convolvulaceae and Poaceae).

It is important to note that the second axis contributes to the ordering of the taxa. Although it is not possible to determine the responsible variable, it is most likely related to the condition of the soil. Species such as *Rhizophora mangle*, *Laguncularia racemosa* and *Avicennia germinans* are all clearly separated. *C. erectus* is associated with mangroves (but only away from the influence of the tide) and is found grouped together with *Metopium brownie* and *B. simaruba*, which prefer well-drained soils but can withstand periodic floods typical of low areas of the peninsula [29].

### Model of the Transfer Function

For the Yucatan Peninsula, no other quantitative reconstructions that use pollen as a proxy exist. This absence rules out a comparison between the statistical values obtained in the construction of the transfer function.

However, the evaluation and selection of a model can be based on a combination of the different parameters. One of these parameters is RMSEP, which in our chosen model took the lowest value (Table 2).

This value indicates the prediction ability of the modern dataset, and therefore, we assert that the calibration model can function as a tool of prediction [2].

It is also necessary to look for a low value for the maximum bias, as it measures systematic differences in the prediction [30]. It is not the lowest value of all the models, but it is much lower in relation to the model of a single component. The medium-high r^2^ value indicates a strong relationship between the inferred values and those observed [2]. The maximum bias and r^2^ statistics were neither the lowest nor the highest, respectively, but the lower RMSEP was certainly important. For a model to be useful, it is necessary that the RMSEP is reduced to at least 5% in relation to the model with the least number of components [31].

Furthermore, it is important to consider that the *p* value must be <0.05 to ensure that the estimates of the model are not just due to chance. In this work, the *p* value of the reconstruction was 0.044. Therefore, we can consider that this value is statistically significant.

**Figure 6:**
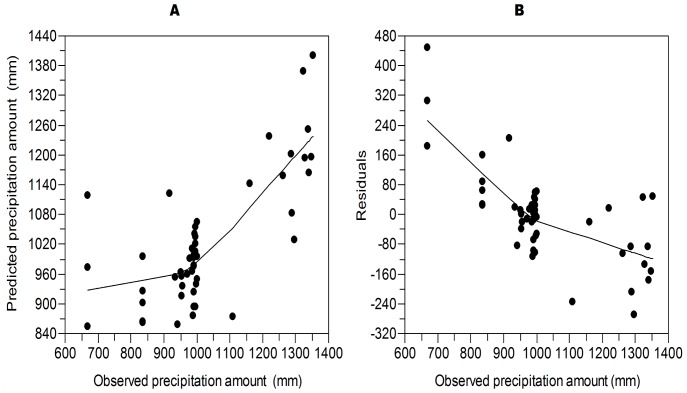
Model diagnostic plots. (A) Relation between estimated (with the transfer function) vs. observed precipitation. (B) Residual relations (observed-estimated) vs. observed precipitation.

The graphs that compare observed values to estimated values (Figure 6a) and the observed values to the residuals (estimated minus observed) (Figure 6b) show that the model predicts values greater than 800 mm relatively well but is less reliable for medium values. However, the low values tend to be overestimated, with differences of at least 160 mm. Overall, the model is generally better with high values than with low values.

### Chronology and Reconstruction of the Vegetation

Research has revealed that the vegetation of the region has been subject to the impact of human activities since early times. The first evidence of maize occurs around 1840 BC. The presence of taxa such as Malvaceae, *Acacia sp.*, Burseraceae, *Ficus* sp. and Fabaceae indicates that, during this stage, the vegetation consisted of secondary forest with Chenopodiaceae, Poaceae, and some cultivated plants. Data from other sites on the Yucatan Peninsula show conditions of low humidity during this stage [6], [26]. For this reason, we may infer that the secondary character of the vegetation is a reaction to the climatic conditions. The presence of Rubiaceae and the increase in abundance of Moraceae indicate that the forest recovered in the late phase of the middle Holocene (1550-1130 BC).

At the beginning of the late Holocene, a reduction in Moraceae and an increase of *Ficus sp.* and Chenopodiaceae are recorded, suggesting disturbed conditions in the region. This disturbance was not related to agricultural activity, as neither *Z. mays* nor elements associated with cultivation, such as *Croton sp*., are present. Circa 950 BC, the vegetation acquired a more open character. This is inferred by the increase in the percentage of *Pinus sp*., which do not form part of the regional vegetation. The presence of this pollen indicates that the arboreal cover was sparse enough to permit the deposition of allochthonous pollen [32]. During this time, the vegetation was a mosaic of low tropical forest, savannas and crops. From 550 BC to 50 AD, the increase in Moraceae; the almost continuous presence of *B. alicastrum*; and the presence of *Pouteria sp.*, Rubiaceae and Sapotaceae, indicate the recovery of the vegetation.

From 50 to 750 AD, the tropical forest suffered the greatest transformation of the studied period. The pollen record shows that tree cover decreased circa 190 AD. This is demonstrated by the reduction of tropical forest taxa (<50%) such as Moraceae (<20%) and the increase of *Pinus sp.* and *Ficus sp*. This change in the vegetation coincides temporally with the Preclassic drought reported in the Maya region [7], [25]–[26], [33]–[35]. In 370 AD, the forest changed to savanna. Pollen from arboreal elements decreased by 15%, while the taxa of disturbance (Poaceae, Chenopodiaceae, Convolvulaceae, *Pinus sp*., among others) reached a total of 84%. During both transformations, agricultural activity was present, but maize pollen also decreased notably during these two events. This suggests that the climate was one of the triggers of the vegetation transformation.

The highest intensity of agricultural activity occurred in 470 AD based on the increase in pollen from maize as well as an increase in Poaceae and *Pinus sp*., among other elements of disturbance. These taxa reach values of 56%, while forest taxa contribute only 29%. This suggests an expansion of savannas and crops but with more arboreal vegetation present than the period of land clearing discussed earlier.

The forest later recovered, and taxa suggest a decrease in the expansion of cultivated and grassland areas, although grasslands continued to be an important component of the vegetation (between 14 and 29%).

The vegetation mosaic of this time was composed of low tropical forest, savannas, crops and mangrove. Evidence of maize was last recorded around 800 AD and was absent in the next analyzed date (860 AD). For this reason, we infer a decrease of agricultural activities and the cultural collapse between 800 and 860 AD.

Circa 1779 AD, the youngest estimated age of this study, forest taxa percentages are the highest of the core. *B. alicastrum* is present continuously and, together with the high percentages of Moraceae and the presence of Anacardiaceae, Rubiaceae and Boraginaceae, suggests more conserved vegetation. There are, however, slight variations which coincide with small oscillations in the curve of *Pinus sp*. and Poaceae, which suggest phases of reduced arboreal cover.

### Quantitative Precipitation Reconstruction

**Figure 7:**
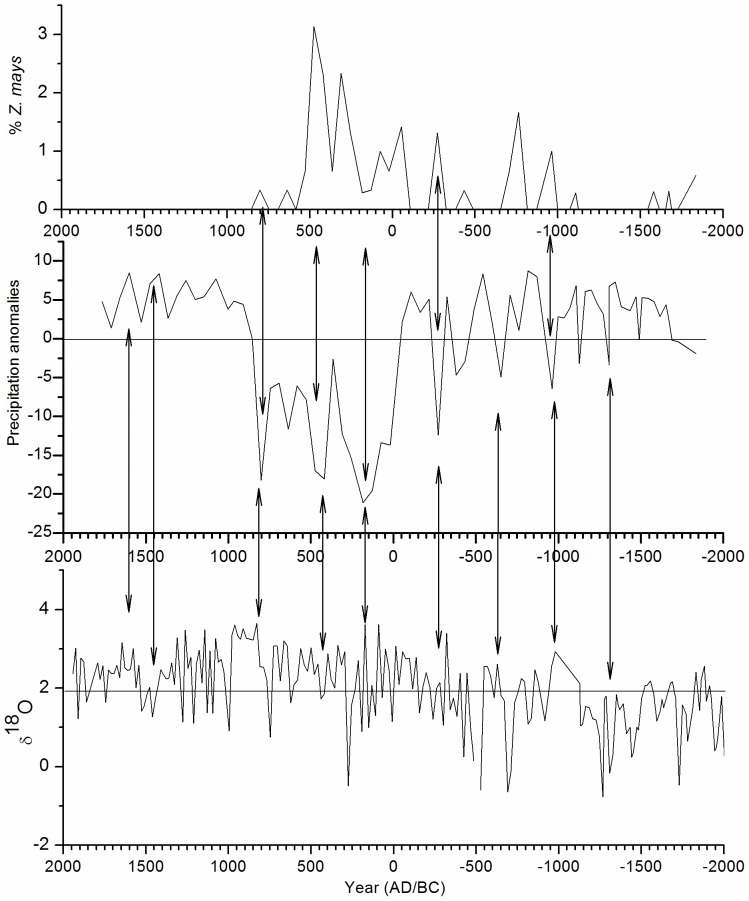
Comparison of records. (A) Record of *Z. mays* of this same study. (B) Anomalies of the inferred precipitation. (C) Record of δ^18^O Lake Chichancanab [6].

The statistical values in the construction of the transfer model indicate that the estimated precipitation from the Ria Lagartos core corresponds to changes in vegetation. However, the vegetation of the region has been impacted since early times by human activity, which may interfere the reliability of the precipitation reconstruction. Nevertheless, as suggested by Leyden et al. [30], even under human influence, the pollen signal maintains a relationship to climatic changes. Therefore, it is indeed useful to compare pollen to the record of precipitation anomalies at Ria Largatos, to the record of *Z. mays* and to the δ^18^O record of Lake Chichancanab [6] (Figure 7). With these comparisons, it is clear that precipitation deficits (droughts) and surpluses (wet phases) do not always correlate with rises and falls in the percentage of *Z. mays*.

In the first phase of the precipitation record, surpluses were more frequent than droughts and longer in duration (Figure 5). Precipitation was 3% higher than the average of the last 3800 years. During this time (1850-1000 BC), isotope values of Lake Chichancanab [6] are lower than the mean, suggesting wet conditions. Changes in the maize record do not match changes in the precipitation record, which suggest that variations in precipitation correspond to climate variability. This phase can be divided into two stages. During the first (1850-1250 BC), precipitation is above average and reaches surpluses of 6.7%. This wet stage can also be observed in a previous study of Ria Largatos [33] and in the Cariaco Basin [34]. During the second stage, droughts occur (precipitation 3.4% below average), but the surpluses are of similar magnitudes to those of the first stage. The beginning (950 BC) of the second phase is marked by a drought where the precipitation decreases by 6.4%. This drought coincides with an increase in the δ^18^O values of Lake Chichancanab [6], Lake Tzib [26] and Lake Punta Laguna [36], along with the decrease in the percentage of forest taxa in the previous study of Ria Largatos [33]. We detect an increase in the maize record. It is probable then that the decrease in precipitation is less than the reconstruction suggests.

The largest surplus (8.75%) is recorded around 320 BC, which agrees with lower δ^18^O values of Lake Chichancanab [6] and with one of the highest percentages of Ti in the last 4000 years in the Cariaco Basin [34]. The recorded increase in precipitation also coincides with an increase in solar radiation [37]. We find that the most intense drought of this phase occurred in 250 BC. The precipitation decreased 12.4%. This drought is apparently a result of the modification of the vegetation by agricultural activities. In Lake Chichancanab, higher δ^18^O values are also observed but do not hold up a reduction of precipitation, nor do we observe such a reduction in Cariaco. However, changes are detected in the records of maize and other elements associated with cultivation, such as *Croton sp.* The second phase ended with an increase in the precipitation (5–6%).

The third phase is clearly differentiated by repeated droughts. During this time, precipitation was below average by an average of 12.6% but at times more than 18%. The first drought lasted from around 130 to 236 AD. During this drought, precipitation dropped by 21%. This phase coincided with what is known as the Preclassic drought reported in various works in the region [26]–[27], [33]–[34]. During this event, important changes in the environment and in Mayan cities occurred, including the collapse of El Mirador (Guatemala), a city that flourished during the Preclassic period [38]. It is important to note that, during this drought, there was no increase in the percentage of maize. The precipitation record confirms that this was a result of the climate and not an artifact of agricultural activity. However, the drought that occurred around 420 AD does coincide with an increase in evidence of maize. In fact, the drought coincides with the highest percentage of maize in the record. Therefore, it is probable that the 18% decrease in precipitation is overestimated. The third drought occurred in 800 AD when precipitation declined 18.2% and, in contrast to the previous drought, no high values of *Z. mays* were observed. This drought coincides with the high δ^18^O values in the study of Lake Chichancanab [6] and decreased precipitation in the Cariaco Basin [28]. The drought also coincides temporally with the second phase of the disintegration of Mayan lands, during which various cities were abandoned. Among these cities were Yaxchilan, Calakmul, Piedras Negras, Copan and Naranjo [38]. Following this drought, maize disappeared from the record and is likely related to the collapse around this time. The collapse is estimated to have occurred between 800 and 860 AD.

Medina-Elizalde proposed that the reduction in precipitation during the final Classic Period was 36–52% based on quantitative precipitation reconstruction using isotopes in stalagmites. The values obtained in this study were more conservative. Nevertheless, it is important to consider that the model constructed tended to overestimate the low values (Figure 6). Therefore, it is likely that droughts were more intense than the values suggest here.

The fourth phase of change (800–1760 AD) is characterized as a wet period. The increase in precipitation was rapid, and the anomaly exceeded an average of 5% with no deficits recorded, despite variable precipitation. Medina-Elizalde [8] suggests that the revitalization of the Puuc region in the north of the peninsula was favored by the precipitation increase, which increased the production of food and could subsequently support a larger population. This event coincides temporally (860–900 AD) with the beginning of this fourth phase, during which the precipitation increased considerably. The period from 900 AD to 1300 AD experienced precipitation surpluses of more than 7.5%. This period coincides with the global event known as the Medieval Climatic Anomaly [39].

Between 1300–1760 AD, precipitation was high overall but also more variable. This period falls into the Little Ice Age (LIA), which was drier than the Medieval Climatic Anomaly but still wetter than the rest of the record. The precipitation reconstruction from the Tzabnabh cave [8] suggests that the LIA was relatively more humid, which agrees with the precipitation record of the present study, yet contrary to earlier findings [34]. However, other studies on fossil pollen on the peninsula have found droughts in this phase [27], [33].

## Conclusions

The calibration of modern fossil pollen with observed precipitation led to the construction of a predictive and reliable model. The model works best with medium values, while low values tend to be overestimated.

The application of a transfer function permitted the reconstruction of precipitation for the last 3800 years. In the area of Ria Lagartos, precipitation anomalies show that the magnitudes of deficits (21%) exceed the surpluses (<9%). Four phases of change have been detected; the third (50 BC – 800 AD) was the driest. The drought related to the abandonment of the Preclassic Period featured an estimated 21% reduction in precipitation, while the drought of the Mayan collapse (800–860 AD) saw a decrease of 18%. The Medieval Climatic Anomaly was a period of positive anomalies (3.8–7.6%), while the Little Ice Age was a more variable period, with reductions but without droughts. Fossil pollen indicates agricultural activity since the early phases (1840), and those activities provoked important changes in the vegetation during the Classic Period. Moreover, data indicate the vulnerability of the vegetation to the combined effect of agriculture and climate. When both agents operate in conjunction, it is possible to cause major transformations in vegetation, such as occurred in 370 AD.

## References

[1] Labeyrie L, Cole J, Alverson K, Stocker T (2003) The History of Climate Dynamics in the Late Quaternary. In Alverson KD, Bradley RS, Pedersen TF, editors. Paleoclimate, global change and the future. Berlin: Springer. 33–61.

[2] Birks HJ (1995) Quantitative palaeoenvironmental reconstructions. In Maddy D, Brew JS, editors. Statistical modelling of Quaternary science data. Technical Guide.

[3] Riehl H (1979) Climate and Weather in the Tropics. London: Academic Press. 611p.

[4] Birks HJ (2005) Quantitative palaeoenvironmental reconstructions from Holocene biological data. In Mackay A, Battarbee R, Birks J, Oldfield F, editors. Global change in the Holocene. Great Britain: Hodder education. 107–123.

[5] Hodell DA, Brenner M, Curtis J (2000) Climate change in the northern American tropics and subtropics since the last ice age. In Lentz DL, editor. Imperfect balance, landscape transformations in the pre-Columbian Americas. New York: Columbia University Press. 13–38.

[6] Hodell DA, Curtis JH, Brenner M (1995) Possible role of climate in the collapse of classic Maya civilization. Nature 375: 391–394.

[7] Curtis JH, Hodell DA, Brenner M (1996) Climate variability on the Yucatan peninsula (Mexico) during the past 3500 years, and implication for Maya cultural evolution. Quatern Res 16: 37–47.

[8] Medina-Elizalde M, Burns SJ, Lea DW, Asmerom Y, van Gunten L, et al (2010) High resolution stalagmite climate record from the Yucatán peninsula spanning the Maya terminal classic period. Earth Planet Sci Lett. 298: 255–262.

[9] Medina-Elizalde M, Rohling EJ (2012) Collapse of Classic Maya Civilization related to modest reduction in precipitation. Sci. 335: 956–959. 10.1126/science.1216629 22363005

[10] Mann ME (2007) Climate over the past two millennia. Annu. Rev. Earth Planet. Sci. 35: 111–136.

[11] CONANP (2007) Programa de conservación y manejo reserva de la biosfera Ría Lagartos. Secretaría de Medio Ambiente y Recursos Naturales. 266p.

[12] CNA (2006) Jefatura de Proyecto de Aguas Superficiales. Subgerencia Regional Técnica. Gerencia Regional Península de Yucatán. Comisión Nacional del Agua. México.

[13] Torrescano-Valle N (2007) Reconstrucción paleoambiental del Holoceno Medio-Tardio en la parte centro-sur de la península de Yucatán, México. Doctoral Thesis. El Colegio de la Frontera Sur.

[14] Erdtman G (1969) Handbook of palynology. An Introduction to the study of Pollen grains and spores. Hafner Publishing Co. 486p.

[15] Palacios-Chavez R, Ludlow-Wiechers B, Villanueva-Gutiérrez R (1991) Flora palinológica de la reserva de la biosfera de Sian Ka’an, Quintana Roo, Mexico. CICRO. 321p.

[16] Correa-Metrio A, Cabrera KR, Bush MB (2010) Quantifying ecological change through discriminant analysis: a paleoecological example from the Peruvian Amazon. J Veg Sci 21: 695–704.

[17] ter Braak CJF (1986) Canonical correspondence analysis: a new eigenvector technique for multivariate direct gradient analysis. Ecology 67: 1167–1179.

[18] ter Braak CJF, Šmilauer P (2002) CANOCO Reference Manual and CANODRAW User’s Guide: Software for Canonical Community Ordination (Version 4.5). Microcomputer Power (Ithaca, New York). 500 p.

[19] Wold S, Ruhe A, Wold H, Dunn WJ (1984) The collinearity problem in linear regression: the partial least squares (PLS) approach to generalized inverses. SIAM J Sci Comput 5: 735–743.

[20] Juggins S (2007) C2 version 1.5 user guide. Software for ecological and palaeoecological data analysis and visualization. Newcastle University, Newcastle upon Tyne, UK. 73p.

[21] Grimm EC (2011) TILIA software version 1.7.16. Illinois State Museum, Research and Collection Center. Springfield USA. Available: http://intra.museum.state.il.us/pub/grimm/tilia/.

[22] Stuiver M, Reimer PJ, Reimer RW (2005) CALIB 5.0 Available: http://calib.qub.ac.uk/calib/.

[23] Reimer PJ, Hughen KA, Guilderson TP, McCormac FG, Baillie MGL, et al (2002) Preliminary Report of the first workshop of the IntCal04 Radiocarbon Calibration/Comparison Working Group. Radiocarbon 44: 653–661.

[24] ter Braak CJF (1987) Unimodal models to relate species to environment. DLO-Agricultural Mathematics Group. 266p.

[25] Torrescano-Valle N, Islebe GA (2006) Tropical forest and mangrove history from south eastern Mexico: a 5000 yr pollen record and implications for sea level rise. Veg Hist Archaeobot 15: 191–195.

[26] Carrillo-Bastos A, Islebe GA, Torrescano-Valle N, González NE (2010) Holocene vegetation and climate history of central Quintana Roo, Yucatan Peninsula, Mexico. Rev Palaeobot Palyno 160: 189–196.

[27] Gutiérrez-Ayala LV, Torrescano Valle N, Islebe GA (2012) Reconstrucción paleoambiental del Holoceno Tardío de la reserva Los Petenes, Península de Yucatán, México. Rev Mex Cienc Geol 29: 749–763.

[28] Leyden BW (2002) Pollen evidence for climatic variability and cultural disturbance in the Maya Lowlands. Anciente Mesoam 13: 85–101.

[29] Pennington DT, Sarukhán J (2005) Árboles tropicales de México: manual para la identificación de las principales especies. Mexico: Universidad Autónoma de México, Fondo de cultura económica. 523 p.

[30] ter Braak CJF, Juggins S (1993) Weigthed averaging partial least squares regression (WA-PLS): an improve method for reconstructing environmental variables from species assemblages. Hydrobiologia 269/270: 485–502.

[31] Birks HJB (1998) Numerical tools in quantitative palaeolimnology-progress, potentialities, and problems. J. Paleolim 20: 307–332.

[32] Islebe GA, Hooghiemstra H, Brenner M, Curtis JH, Hodell DA (1996) A Holocene vegetation history from lowland Guatemala. The Holocene 6: 265–271.

[33] Aragón-Moreno AA, Islebe GA, Torrescano-Valle N (2012) A ∼3800-yr, high-resolution record of vegetation and climate change on the north coast of the Yucatan Peninsula. Rev Palaeobot Palynol 178: 35–42.

[34] Haug GH, Gunther D, Peterson LC, Sigman DM, Hughen KA, et al (2003) Climate and the Collapse of Maya Civilization. Sci 299: 1731–1735. 10.1126/science.1080444 12637744

[35] Hodell DA, Brenner M, Curtis JH, Guilderson T (2001) Solar Forcing of Drought Frequency in the Maya Lowlands. Sci 292: 367–1370. 10.1126/science.1057759 11359010

[36] Hodell DA, Brenner M, Curtis JH (2007) Climate and cultural history of the Northeastern Yucatan Peninsula, Quintana Roo, Mexico. Climatic Change 83; 215–240.

[37] Steinhilber JAA, Beer J (2008) Solar modulation during the Holocene. Astra 4: 1–6.

[38] Gill RB (2008) Las grandes sequías mayas, agua, vida y muerte. México Distrito Federal: Fondo de cultura económica. 561 p.

[39] Mann ME (2002) Medieval climatic optimum. In: MacCracken MC, Perry JS, editors. Encyclopedia of global environmental change. Chichester: Jonh Wiley and Sons 514–516.

